# Hydrogel of HEMA, NVP, and Morpholine-Derivative Copolymer for Sulfate Ion Adsorption: Behaviors and Mechanisms

**DOI:** 10.3390/molecules28030984

**Published:** 2023-01-18

**Authors:** Jing Zhao, Haitao Liu, Wenwen Chen, Yu Jian, Guoyong Zeng, Zhenyu Wang

**Affiliations:** 1 Laboratory of Organic Solid Waste Treatment and Recycling, College of Materials Science and Engineering, Henan Institute of Technology, Xinxiang 453003, China; 2 College of Chemistry and Materials Engineering, Wenzhou University, Wenzhou 325035, China; 3 Department of Chemistry, Lishui University, Lishui 323000, China; 4 Department of Environmental Engineering, College of Ecology, Lishui University, Lishui 323000, China

**Keywords:** sulfate ion, hydrogel, morpholine groups, adsorption isotherms

## Abstract

SO_4_^2−^-containing compounds are widely present in wastewater generated from various industries and mining industries, such as slag leachate, pulp and paper wastewater, modified starch wastewater, etc. When the concentration of SO_4_^2−^ is too high, it will not only be corrosive to metal equipment but also accumulate in the environmental media. Based on this, a novel cationic hydrogel HNM was synthesized in this study by introducing morpholine groups into the conventional hydrogel HEMA–NVP system for the adsorption of SO_4_^2−^ in aqueous solutions. Characterizations by Fourier transform infrared (FTIR) spectroscopy and X-ray photoelectron spectroscopy (XPS) indicated that morpholine groups had been introduced into the as-synthesizedhydrogels. The scanning electron microscope (SEM) characterization results show that the introduction of morpholine groups changed the surface of the hydrogel from micron-scale wrinkles to nanoscale gaps, increasing the contact area with the solution. The results of static water contact angle (WCA), equilibrium water content (EWC), and SO_4_^2−^ adsorption capacity show that the introduction of morpholine groups not only further improved the equilibrium water content and hydrophilicity of the hydrogel but also greatly improved the SO_4_^2−^ adsorption capacity of the hydrogel, with the maximum SO_4_^2−^ adsorption amount of 21.59 mg/g, which was much higher than that of the hydrogel without morpholine groups of 5.15 mg/g. Further studies found that the adsorption of SO_4_^2−^ on the hydrogel HNM was pH-dependent, and acidic conditions were favorable for the adsorption. Therefore, the introduction of morpholine groups greatly enhanced the ability of conventional HEMA–NVP hydrogels to remove SO_4_^2−^ from aqueous solutions.

## 1. Introduction

Sulfate ions are widely distributed in surface water, groundwater, and industrial wastewater, such as acid mine, pharmaceutical, printing, and dyeing wastewater [1]. Although sulfate ions are usually considered nontoxic, they are potentially harmful to organisms and the environment. High concentrations of sulfate ions in water can lead to an imbalance in the natural sulfur cycle in ecosystems and can be hazardous to human health when ingested in excess over time [2,3]. In addition, sulfate ions in the environment can also cause serious damage to buildings. For example, the hydration products in concrete structures in buildings react with sulfate ions to form gypsum and calcium alumina, leading to the expansion and cracking of concrete structures, reduction of strength, and ultimately structural damage [4,5]. Therefore, efforts to reduce or eliminate sulfate ions in the environment are important.

Currently, the main techniques for eliminating sulfate ions include chemical precipitation, ion exchange, biological treatment, adsorption, and membrane filtration [6]. However, chemical precipitation and ion exchange methods are prone to problems, such as water deterioration and soil acidification, and biological treatment and membrane filtration treatment are more expensive, so these methods are somewhat limited in practical application [7]. Adsorption methods have the advantages of high removal efficiency, stable performance, simple operation, and high cost-effectiveness and have attracted increasing research attention worldwide [8,9]. Currently, commonly used adsorption materials include metal–organic frameworks (MOFs) [10], carbon-based materials [11], and zeolites [12], but the performance of these materials needs further improvement. For example, carbon-based materials and zeolites have limitations with low adsorption capacity and weak selectivity and stability [13]. Although MOF materials have good absorption properties, the disadvantages, such as complicated preparation, high energy cost, and unstable aqueous solution, limit their potential applications [14]. Therefore, exploring new adsorbent materials with high performance, easy operation, and low cost has become an attractive research topic.

In recent years, hydrogels have attracted much attention because of their unique physicochemical properties. Hydrogels have a three-dimensional network structure, which can absorb large amounts of water or other aqueous fluids and maintain their integrity by physical (ionic, hydrogen, or van der Waals bonds) or chemical (covalent bonds) cross-linking [15]. Moreover, hydrogels can adsorb and trap ions and molecules through a porous network structure capable of physicochemical interactions with molecules and ions in aqueous fluids. In addition, hydrogels respond to environmental stimuli (pH, salt concentration, temperature, etc.) under weak changes in the stimuli [16,17,18]. Therefore, their wide application in biotechnology, medicine, agriculture, food industry, and water purification has attracted great interest in the last decades [19,20]. However, pure hydrogels have no specific sites and poor adsorption of sulfate ions.

The adsorption performance of hydrogels for anions can be greatly improved by incorporating various organic materials and metal ions into the hydrogels. For example, Fang prepared a new high-capacity cationic hydrogel adsorbent from an aqueous medium by condensation with triethylenetetramine, acetone, and formaldehyde. The hydrogel has a high cationic charge density and abundant functional groups and has a high adsorption uptake rate for anionic dyes [21]. Morpholine is a six-membered cyclic compound containing nitrogen and oxygen atoms. Due to their biological and pharmacological properties, morpholines are used in many applications, such as antioxidant [22] and anti-inflammatory [23]. In addition, morpholine groups have a positive charge, and their electrochemical properties are remarkable, and there are many studies on the electrochemical applications of compounds containing morpholine groups [24,25,26,27]. However, to our knowledge, there is no literature on the synthesis, characterization, and application of morpholine groups in hydrogel materials for anion adsorption.

In this paper, copolymer hydrogels with different contents of 2-morpholine ethanol monomer (HEMA–NVP–Mor) were prepared by introducing 2-morpholine ethanol as a copolymer monomer into the conventional HEMA–NVP copolymer hydrogel system. The structural morphology, surface chemistry, and thermal stability of the prepared hydrogels were characterized using FTIR, SEM, XPS, TGA, and other analytical methods. The effects of the content of 2-morpholine ethanol monomer, the initial pH value of the solution, the adsorption concentration, and the adsorption time on the adsorption performance of the materials were further investigated. The experimental results show that the introduction of morpholine groups increased the specific surface area of the hydrogel and improved the adsorption capacity of sulfate ions. In addition, the adsorption capacity of the polymeric materials for sulfate at different pH values was also investigated. This study not only promotes the research of using modified hydrogels to treat sulfate ions in wastewater but also provides ideas for the removal of other anionic pollutants.

## 2. Results and Discussion

**Figure 1:**
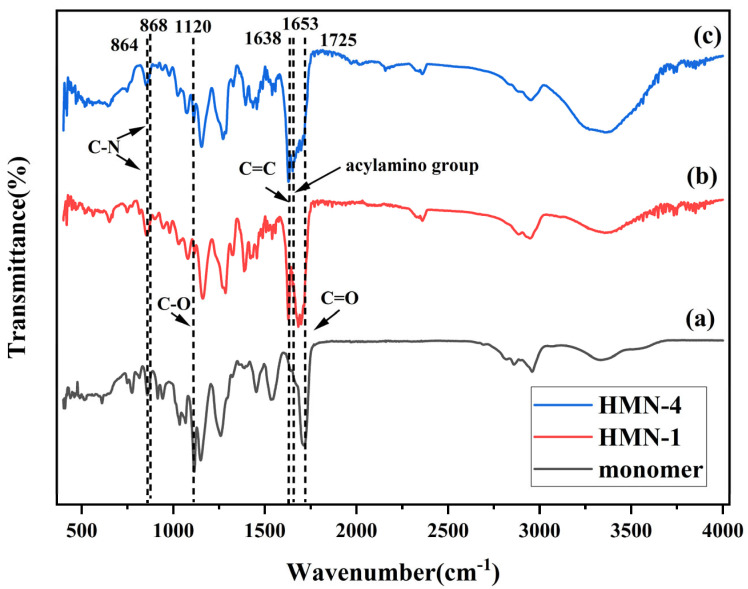
FTIR spectra of (**a**) monomer, (**b**) HMN-1, and (**c**) HMN-4.

**Figure 2:**
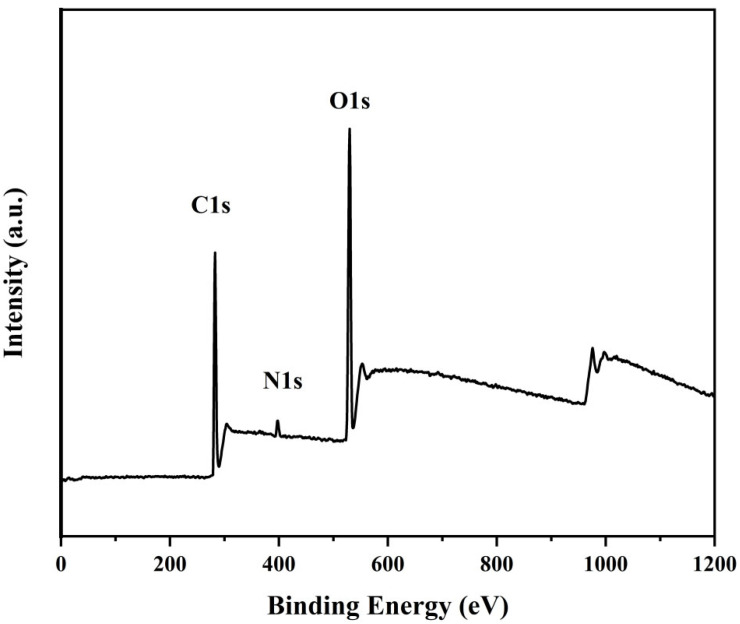
XPS spectra of the hydrogel HMN-4.

To identify the functional groups and their chemical composition characterization, FTIR was used for the analysis. The infrared spectra of MCAEM monomer, HMN-1, and HMN-4 were tested and analyzed as shown in Figure 1. In curve (a), 864 cm^−1^ and 868 cm^−1^ are the vibrational peaks of C–N on the morpholine ring in 2-morpholine ethanol; 1625 cm^−1^ and 1120 cm^−1^ are the vibrational peaks of C=O and C–O of the ester group on HEMA, and the characteristic peak at 1653 cm^−1^ is attributed to the generated imino group. In curve (b), the characteristic peaks at 1725 and 1653 cm^−1^ are attributed to the ester group in HEMA and the acyl group in NVP, and the 1638 cm^−1^ vibrational peak of C=C has disappeared, indicating that HEMA and NVP have been polymerized into hydrogels. In curve (c), compared with curve (b), a vibrational peak of 868 cm^−1^ (C–N of the morpholine ring in MCAEM) appears, indicating that MCAEM has successfully polymerized with HEMA and NVP. The chemical properties of the surface of the hydrogel HMN-4 sample were further analyzed by XPS, and the test results are shown in Figure 2. The XPS broad spectrum showed three characteristic signal peaks of Cls (283 eV), O1s (530 eV), and N1s (398 eV) on the surface of the hydrogel, indicating the presence of C, O, and N elements on the surface of the hydrogel, which is consistent with the FTIR test results.

**Figure 3:**
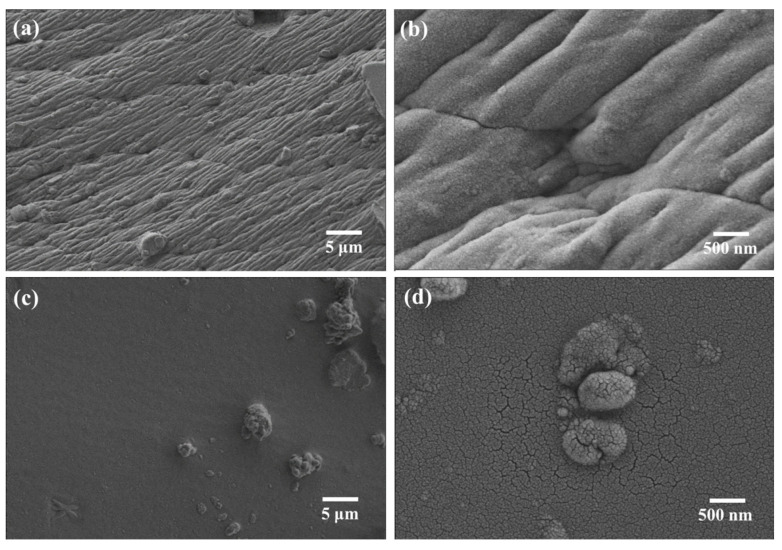
SEM images of (**a,b**) HMN-1 and (**c,d**) HMN-5.

The surface morphologies of the synthesized hydrogel materials HNM-1 and HNM-4 were characterized by SEM, and the results are shown in Figure 3. As shown in Figure 3a, the surface of HNM-1 exhibits a micron-sized furrow morphology, and some granular bumps exist on the surface. When the surface of HNM-1 was further enlarged, it showed an obviously folded morphology (as shown in Figure 3b). While the surface of HNM-4 has some granular bumps, it shows a smoother and flatter shape overall, as shown in Figure 3c. When the surface of HNM-4 is further magnified, there are nanoscale gaps on the surface of HNM-4, and these gaps are beneficial to increase the contact area of the hydrogel. As seen in the figure, the addition of the MCAEM monomer enhances the flatness of the surface of the hydrogel material, reduces the roughness of the surface, and improves the contact area with the solution.

**Figure 4:**
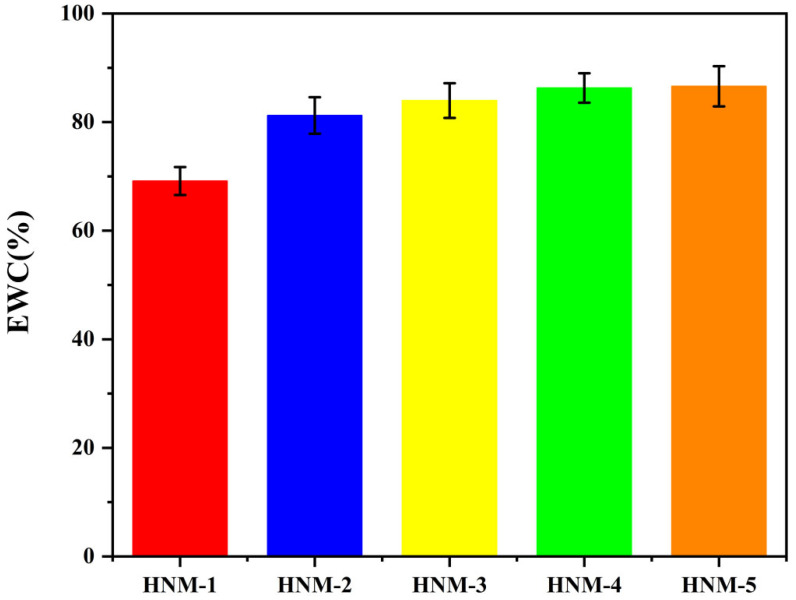
Equilibrium water content of HNM-1, HNM-2, HNM-3, HNM-4, and HNM-5.

The effect of copolymeric monomers on the EWC of hydrogels is shown in Figure 4. As seen in the figure, as the content of the MCAEM monomer increased, the EWC value of the synthesized hydrogel material then increased until saturation, from 69.15% for HNM-1 to 86.59 % for HNM-5. This is mainly because the MCAEM monomers are hydrophilic monomers, so as the proportion of MCAEM in the synthesized hydrogel increases, the ability of the material to adsorb water molecules inside the material increases, leading to an increase in the EWC value of the material. When the water content inside the material reaches a certain value, the ability to accommodate water molecules in the network structure of the material reaches saturation, so the EWC value of the hydrogel material no longer increases as the proportion of MCAEM in the synthetic hydrogel increases further.

**Figure 5:**
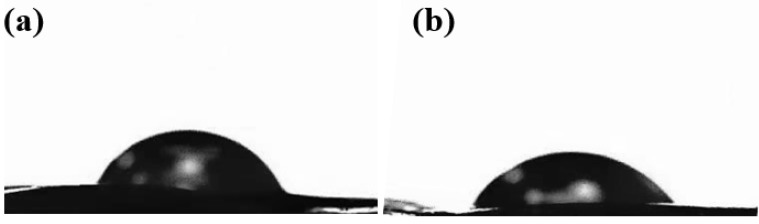
Static contact angle of (**a**) HNM-1 and (**b**) HNM-4 is 65.49° and 51.96°, respectively.

The measured static contact angles of HNM-1 and HNM-4 are shown in Figure 5. The WCA of HNM-1 without the MCAEM monomer was 65.49° (Figure 5a), which indicates the good hydrophilicity of the unmodified hydrogel. In contrast, the WCA of the HNM-4 hydrogel surface decreased to 51.96° (Figure 5b), which indicated the enhanced hydrophilicity of the synthetic hydrogel with the addition of the MCAEM monomer and the enhanced wettability of the material surface. The static contact angle measurements were consistent with the EWC-worthy measurements.

**Figure 6:**
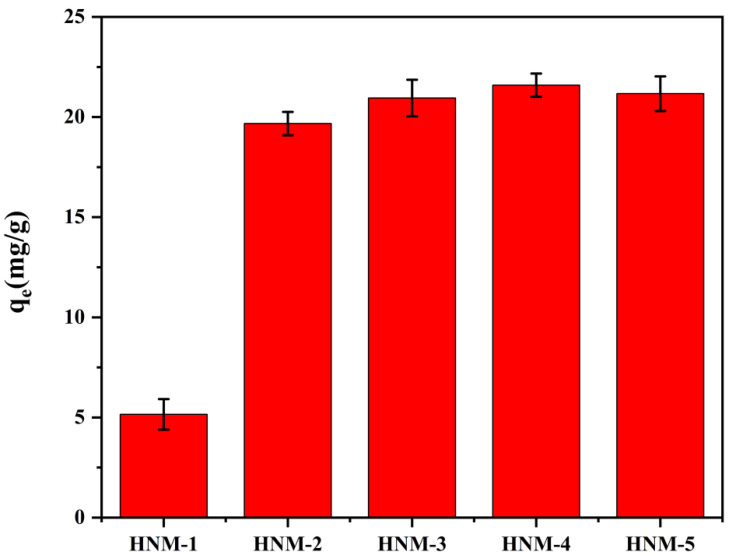
Adsorption capacities of HNM-1, HNM-2, HNM-3, HNM-4, and HNM-5 for SO_4_^2−^.

The effect of the variation of the MCAEM content in the polymer on the adsorption effect of the hydrogels was investigated, and the results are shown in Figure 6. All four hydrogels with the added MCAEM monomer showed good adsorption capacity for sulfate ions. Among them, HNM-4 showed the best adsorption effect with the maximum adsorption amount of 21.59 mg/g for sulfate, while HNM-1, which did not contain the MCAEM monomer, showed the maximum adsorption amount of only 5.15 mg/g. The results may be due to the presence of the MCAEM branched chains in the polymer network, which provided positively charged adsorption sites and easier adsorption of sulfate ions into the hydrogel network. The decrease in the adsorption amount after reaching the maximum value on the HNM-4 surface may be due to the large number of the MCAEM branched chains crossing with other branched chains, reducing the space of pores in the three-dimensional network of the hydrogel, thus leading to a decrease in the maximum adsorption capacity of the hydrogel for sulfate ions.

**Figure 7:**
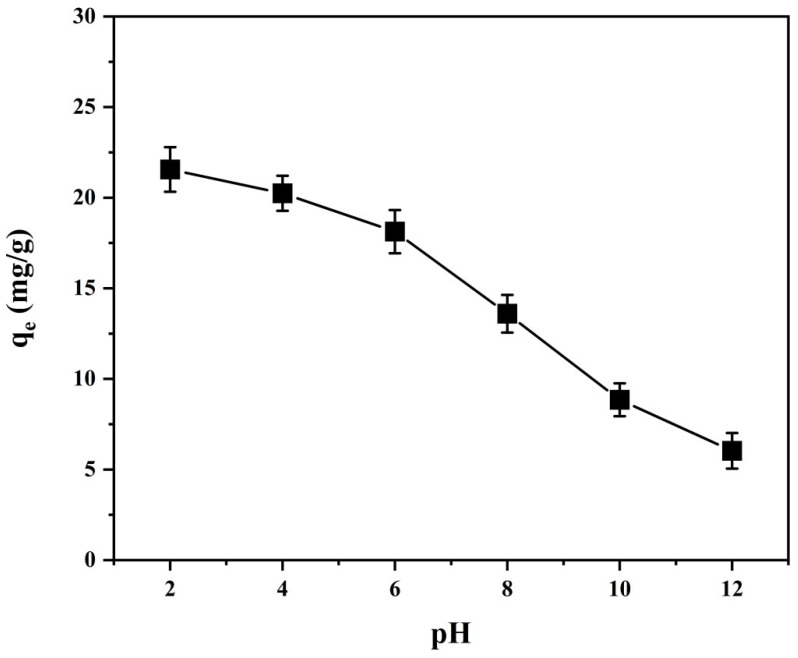
Effect of pH on the SO_4_^2−^ adsorption capacity of HNM-4.

The adsorption performance of a hydrogel adsorbent depends largely on the pH of the solution, because pH can affect the surface charge of the adsorbent and thus the adsorption capacity of the hydrogel. In the presence of hydrogels, the initial pH of the solution changes, and when the difference between the equilibrium pH and the initial pH is zero, the pH of the solution is called the pHPZC at the point of zero charge. Therefore, when the solution pH is equal to the pHPZC, the hydrogel will remain neutral, as shown in Table 1, where the pHPZC values of HNM-1 to HNM-5 were tested. The sulfate ion adsorption capacity of the hydrogel HNM-4 was further tested at a different initial pH, and the results are shown in Figure 7. HNM-4 hydrogel showed a pH-dependent adsorption behavior, and the adsorption capacity is greater at a low pH and decreases with increasing pH. When the solution pH is less than pHPZC, the surface of the hydrogel becomes positively charged, and SO_4_^2−^ is easily captured by the hydrogel through electrostatic attraction. When the pH > pHPZC, the charge on the hydrogel surface reverses from positive to negative with increasing pH, leading to an increase in electrostatic repulsion and a decrease in SO_4_^2−^ uptake. In addition, at a high pH, the large amount of OH- ions present in the solution compete with sulfate ions for the active adsorption sites on the HNM-4 surface, which also leads to a decrease in the sulfate adsorption capacity. Therefore, the adsorption capacity of sulfate ions is higher in acidic environments.

**Figure 8:**
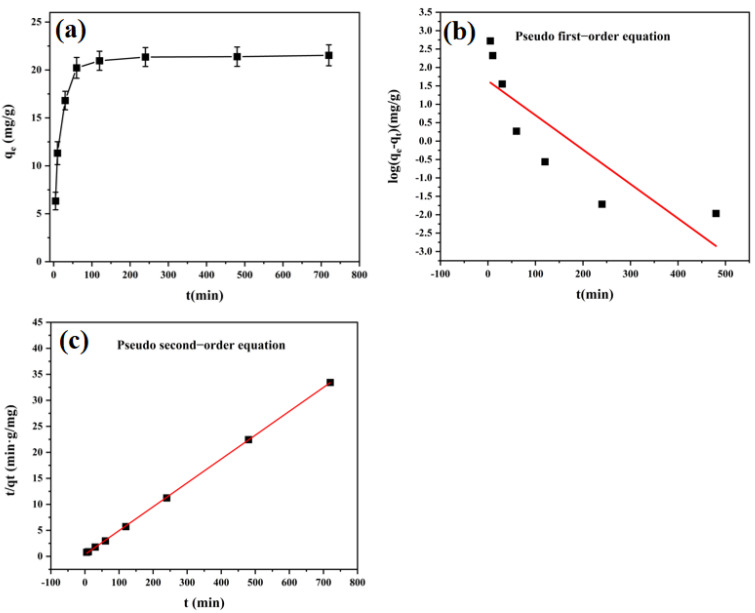
(**a**) Adsorption isotherm, (**b**) pseudo−first−order model, and (**c**) pseudo−second−order model of HNM-4.

The kinetics of the adsorption of hydrogel HNM-4 were studied and analyzed. The sulfate ion adsorption process can be divided into two stages: the rapid increase stage and the near equilibrium stage. The equilibrium time of sulfate ion adsorption was about 240 min, and the equilibrium adsorption amount was 21.53 mg/g, as shown in Figure 8a. In contrast, ion exchange resins were often used to adsorb sulfate ions. However, its affinity for water was far less than that of hydrogels. Therefore, it was difficult for sulfate ions to diffuse in the adsorbent with water, resulting in poor adsorption capacity [28,29]. At the beginning of 60 min, the adsorption amount of SO_4_^2−^ increased rapidly with time, and the adsorption rate was very fast, because the active site (-OH) on the surface of HNM-4 was occupied rapidly by SO_4_^2−^ in water, and this was the rapid increase stage. A total of 120 min later, the active site on the surface of HNM-4 gradually approached saturation, and the adsorption rate decreased, resulting in the SO_4_^2−^ adsorption capacity After 120 min, the active sites on the HNM-4 surface gradually approached saturation, and the adsorption rate decreased, resulting in the slow growth of SO_4_^2−^ adsorption capacity, and the kinetic curve tends to level off, which is close to the equilibrium stage. To further explore the kinetic characteristics of adsorption, pseudo-first-order and pseudo-second-order models were applied to fit the adsorption kinetic data. The kinetic equations are shown below.

$$\mathrm{Pseudo}\text{-}\mathrm{first}\text{-}\mathrm{order}\;\mathrm{equation}:\frac{dq_{t}}{dt}=k_{1}\left(q_{e}-q_{t}\right)$$

$$\mathrm{Pseudo}\text{-}\mathrm{second}\text{-}\mathrm{order}\;\mathrm{equation}:\;\frac{dq_{t}}{dt}=k_{2}{\left(q_{e}-q_{t}\right)}^{2}$$

where q_t_ (mg/g) and q_e_ (mg/g) are the adsorption amounts of sulfate ions at time t and equilibrium, respectively. k_1_ (min^−1^) and k_2_ (g/mg/min) are the rate constants of the pseudo-first-order and pseudo-second-order models, respectively. The analytical results of the pseudo−first−order and pseudo−second−order models are shown in Figure 8b and Figure 8c, respectively, and the relevant parameters are listed in Table 2. The fitting results show that the kinetics of HNM-4 adsorption on sulfate ions follow the pseudo-second-order model more, and its correlation coefficient (R_2_) is close to 1.0, which indicates that the rate control of the adsorption process is a chemical reaction process.

**Figure 9:**
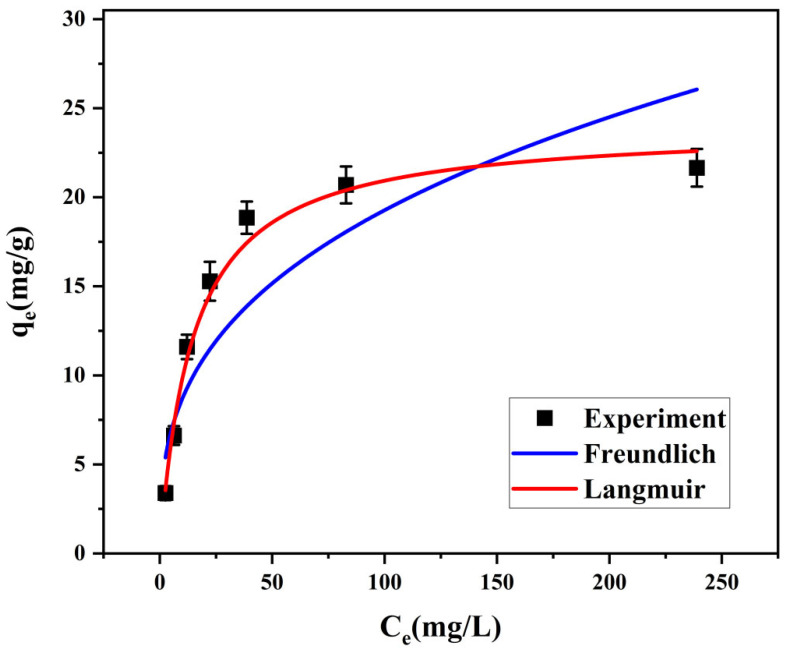
Adsorption isotherm, Langmuir isotherm, and Freundlich of HNM-4.

The equilibrium adsorption of HNM-4 was studied under different initial concentrations of sulfate ions, and the results are shown in Figure 9. The results show that the initial concentration of sulfate ions plays an important role in the uptake of sulfate ions by the hydrogel. It is clear from Figure 9 that the equilibrium sulfate ion adsorption increases as the initial concentration of sulfate ion increases from 50 to 250 mg/L. The higher the initial concentration of sulfate ions, the higher the uptake of sulfate ions, which is due to the strong electrostatic interaction between the anionic sulfate ion group and the hydrogel with acidic pH. This is because the higher concentration of the solution provides a stronger driving force for the mass transfer of sulfate ions between the aqueous–solid phase. Therefore, a high concentration of sulfate ion solution facilitates the adsorption uptake by the adsorbent. Such behavior has been reported in the literature [30,31].

The adsorption process of sulfate ions was further investigated by fitting the results of the HNM-4 equilibrium adsorption tests for different initial concentrations of sulfate ions using the Freundlich as well as the Langmuir adsorption isotherm curves. The Freundlich and Langmuir equations are shown below.

$$\mathrm{Langmuir}\;\mathrm{model}:\;q_{e}=\frac{Q_{max}K_{l}C_{e}}{1+K_{l}C_{e}}\;\mathrm{Freundlich}\;\mathrm{model}:\;q_{e}=K_{f}{\left(C_{e}\right)}^{n}$$

where Q_e_ (mg/g) and C_e_ (mg/L) are the equilibrium adsorption amount and sulfate ion concentration at equilibrium, respectively, Q_max_ (mg/g) is the maximum adsorption capacity, K_l_ (L/mg) and K_f_ ((mg/g)(L/mg)^1/n^) are the Langmuir and Freundlich constants, respectively, and n is the Freundlich linear constant.

Based on the comparison of the calculated correlation coefficient (R_2_) values, the Langmuir model is more consistent with the experimental data, which indicates that monolayer adsorption may have occurred. The adsorption of HNM-4 on sulfate ions increased with increasing concentration of sulfate ions until the adsorption equilibrium, which is because the binding probability of HNM-4 adsorption sites increased with the initial concentration of sulfate ions. The adsorption amount reached the maximum value at the concentration of 150–200 mg/L of sulfate ions, and the adsorption amount gradually reached equilibrium at the concentration of 200–250 mg/L of sulfate ions. As shown in Table 3, the fitting coefficient R_2_ of the Langmuir isotherm model was the largest, so the adsorption reaction process was more suitable to be described by the Langmuir isotherm model, which indicated that the adsorption of sulfate ions by HNM-4 belonged to monolayer adsorption.

**Figure 10:**
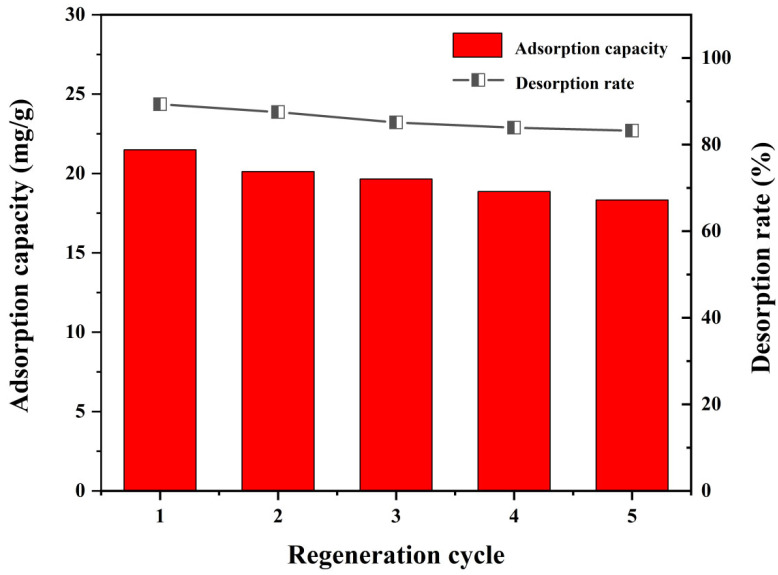
Variation of adsorption capacity and desorption rate by HNM-4 in regeneration cycle.

An important property of adsorbent is its reusability. Figure 10 shows the variation trend of SO_4_^2−^ adsorption capacity and desorption rate in the regeneration cycle of HNM-4. As shown in the figure, after five adsorption–desorption cycles, the adsorption capacity of SO_4_^2−^ of HNM-4 decreased from 21.49 mg/g to 18.33mg/g, and the desorption rate decreased from 89% to 83%. The adsorption capacity and desorption rate of the regenerated HNM-4 decreased, but the desorption rate remained above 80%, which also indicated the potential of the reuse of the adsorbent HNM-4. One of the reasons for the decreased desorption rate of the adsorbents is that the bound SO_4_^2−^ cannot be desorbed, resulting in the inability to continue adsorption of SO_4_^2−^ [32]. Therefore, the adsorption site of the hydrogel adsorbent was increased to improve the adsorption capacity after regeneration. In addition, the morpholine group was polymerized in the main chain of hydrogel, which avoids the decrease in SO_4_^2−^ adsorption capacity caused by the loss of active sites. Therefore, the hydrogel prepared in this study has excellent reusability.

## 3. Experimental

### 3.1. Materials

**Scheme 1:**
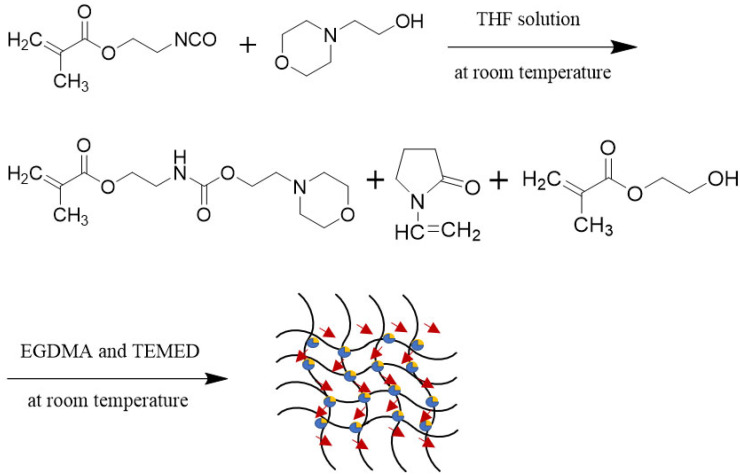
Schematic diagram of the synthesis of copolymer hydrogels.

2-hydroxyethyl methacrylate (HEMA), N-vinylpyrrolidone (NVP), ethylene glycol dimethacrylate (EGDMA), potassium persulfate (KPS), N,N,N′,N′-tetramethyl ethylenediamine (TEMED), 2-morpholine ethanol, isocyanoethyl methacrylate, and ethyl acetate were purchased from Shanghai Mac Ltd. All the aqueous solutions were prepared to use laboratory-prepared deionized water (0.055 μΩ). HNM Hydrogels were firstly prepared as a novel adsorbent for adsorption of SO_4_^2−^, as shown in Scheme 1.

### 3.2. Preparation of 2-(((2-Morpholineethoxy)carbonyl)amino)ethyl Methacrylate

The reaction was carried out by adding 5.33 g of 2-morpholine ethanol into a single-necked round-bottom flask, then adding 28 g of tetrahydrofuran to dissolve, and then adding 6.21 g of isocyanoethyl methacrylate, inserting a condenser tube, and stirring uniformly for 24 h at room temperature. After the reaction, the solution was recrystallized by adding petroleum ether, and the mixed liquid was poured into a filtering flask and filtered by rinsing with 1:1 ethyl acetate petroleum ether three times, and finally 2-((2-Morpholineethoxy)carbonyl)amino)ethyl methacrylate (MCAEM) was obtained by drying at room temperature.

### 3.3. Preparation of HNM Hydrogels

At room temperature, a certain amount of HEMA, NVP, and MCAEM were dissolved in deionized water to obtain the initial mixed solution, and the specific proportions of monomers are detailed in Table 4. Then, the initial mixed solution was transferred to an ice water bath, and EGDMA, KPS, and TEMED were added sequentially, with the amounts of EGDMA and KPS being 0.50 and 0.10 wt% of the total amount of monomers in the initial mixed solution, respectively, while the molar ratio of TEMED to KPS was 0.215. Then, the final mixed solution was slowly poured into a glass dish and subjected to free radical copolymerization at room temperature. The reaction was stopped after 24 h. The resulting hydrogel was immersed in deionized water, and the deionized water was changed every 12 h for 8 days to remove unreacted monomers. The prepared copolymer hydrogels were hydrogel sheets with a thickness of about 0.5 cm.

### 3.4. Characterization

The FTIR spectra of the hydrogels were tested on an EQUINOX55 FTIR spectrometer (Bruker, Rheinstetten, Germany). The surface chemistry of the hydrogel surface was tested by XPS using an ESCA 5600 spectrometer (Perkin Elmer, Waltham, MA, USA) with an MgKa X-ray source (1253.6 eV), and the spectra were obtained at a take-off angle of 0°, obtaining measured spectra of the samples from 0 to 1200 eV. Scanning electron microscope (JEOL JSM-7800F, Tokyo, Japan) at an acceleration voltage of 10.0 kV was used to observe the surface morphology of the samples. The static contact angle of water droplets on the surface of hydrogel sheets was measured at 25 °C using a contact angle goniometer (OCA 20, Dataphysics, Stuttgart, Germany). A 2 μL drop of water was carefully placed on the surface of the dissolved hydrogel sheet and then observed with an optical microscope to determine the static water contact angle of the test surface. The equilibrium water content of the hydrogels was determined at room temperature using the weight method. The hydrogels were placed in deionized water and absorbed, swelled, and weighed at specified time intervals until equilibrium was reached. Excess water was wiped off the surface of the hydrogels with wet filter paper, and the equilibrium wet weight was recorded. Subsequently, the hydrogel samples were dried to constant weight in a vacuum at 80 °C and weighed. The EWC of the hydrogel was calculated by the following equation:

$$\mathrm{EWC}=\frac{\left(m_{e}-m_{d}\right)}{m_{e}}\times 100\%$$

where m_e_ is the saturated constant weight, and m_d_ is the dry constant weight.

A total of 1.000 g of analytical grade anhydrous sodium sulfate was weighed and then diluted to 1000 mL in a standard measuring flask to prepare a standard sulfate solution of 1000 ppm. A series of experimental sulfate solutions were prepared by diluting 5 mL, 10 mL, 15 mL, 20 mL, 25 mL, and 30 mL of the standard sulfate solution in six 100 mL volumetric flasks.

The hydrogels were tested for their sulfate ion adsorption capacity. A 0.5 cm × 0.5 cm hydrogel was added to 50 mL of 1000 mg/L SO_4_^2−^ solution at room temperature and adsorbed in a constant temperature water bath at 25 °C with a shaking rate of 120 rpm for 3 h. The equilibrium concentration of sulfate in the water after adsorption experiments was determined using a UV-Vis spectrometer (PerkinElmer Lambda 35, Perkin Elmer, Waltham, MA, USA). The measurements were carried out at a maximum wavelength (λmax) of 420 nm [33,34].

The equilibrium adsorption amount (q_e_) of the hydrogel was calculated as follows:

$$q_{e}=\frac{\left(C_{0}-C_{e}\right)\times V}{m}$$

where C_0_ (mg/L) and C_e_ (mg/L) are the initial and equilibrium concentrations of sulfate ions, respectively, m (g) is the mass of the hydrogel, and V (L) is the volume of the sulfate solution.

After ion adsorption, desorption experiments were carried out by replacing the equilibrium solutions with the same volume of 0.01 mol /L NaOH and shaking for 120 min. Sulfate concentrations in the extracts were measured by UV-spectrophotometeric method. Repeat the adsorption–desorption process for 5 times.

The desorption rate was calculated as follows:

$$\eta =\frac{C_{\mathrm{des}}V}{{\mathrm{mq}}_{e}}\times 100\%$$

where C_des_ (mg/L) are the desorption concentrations of sulfate ions, m (g) is the mass of the hydrogel, V (L) is the volume of the KCl solution, and q_e_ (mg/g) is the equilibrium adsorption.

The point of zero charge (PZC) of the hydrogel was determined. A total of 2 g of hydrogel was stirred with 100 mL of 0.1 M potassium nitrate solution in a closed Erlenmeyer flask at 25 °C for 24 h to reach equilibrium. The initial pH (phi) was adjusted to a value between 2 and 12 by adding 0.1 M HNO_3_ or 0.1 M KOH solution. A plot of the final pH (phf) versus phi value was used to determine the PZC of the hydrogel.

The equilibrium adsorption of sulfate ions on the hydrogels was investigated at different pHs. The equilibrium adsorption amounts were measured and calculated after adding 0.5 cm × 0.5 cm hydrogels to 50 mL of 1000 mg/L SO_4_^2−^ solutions of different pH at room temperature and adsorbed in a constant temperature water bath at 25°C with a shaking rate of 120 rpm for 5 h. The pH values were adjusted by adding 0.1 mol/L HCl and NaOH solutions (pH = 2, 4, 6, 8 10, and 12). The equilibrium concentration of sulfate in water after the reaction was determined using a UV-Vis spectrometer.

The adsorption kinetics of the prepared obtained hydrogels were studied. A 0.5 cm × 0.5 cm hydrogel was added to 50 mL of 300 mg/L SO_4_^2−^ under certain pH conditions. The adsorption reaction was carried out in a constant temperature water bath at 25 °C with a shaking rate of 120 rpm, and the adsorption amount was measured by aspirating the solution at certain times (t = 5, 10, 30, 60, 120, 240, 480, and 720 min). The 0.5 cm × 0.5 cm hydrogels were added to 50 mL of different concentrations of SO_4_^2−^ solution (C_0_ from 5 to 300 mg/L) under optimum pH conditions. The adsorption was carried out in a constant temperature water bath at 25 °C with a shaking rate of 120 rpm for 3 h. The equilibrium concentration of sulfate in the water after the reaction was determined using a UV-Vis spectrometer.

## 4. Conclusions

In this study, a novel cationic MCAEM monomer was synthesized in one step from 2-morpholine ethanol, and a new cationic hydrogel was further prepared by polymerization with HEMA and NVP radicals to obtain a novel cationic hydrogel. The hydrogels were characterized by FTIR and XPS, and the results show that morpholine groups had been introduced into the hydrogels. The scanning electron microscopy characterization results show that when the morpholine group was introduced into the hydrogel, the surface of the hydrogel changed from micron-scale folds to nanoscale gaps, which increased the contact area with the solution. The EWC and contact angle of the hydrogels were tested, and it was found that as the content of the hydrophobic monomer MCAEM in the hydrogels increased, the equilibrium water content of the hydrogels increased, the static contact angle decreased, and the hydrophilicity increased. The factors affecting the adsorption performance of the hydrogels, such as the monomeric MCAEM content and the pH of the medium, were further investigated. It was found that the introduction of the MCAEM monomer in the hydrogel effectively improved the adsorption capacity of the hydrogel for sulfate ions, but the excessive content caused a slight decrease in the adsorption capacity. Moreover, the adsorption capacity of the hydrogel decreased as the solution pH value increased. Therefore, the adsorption capacity of the synthetic hydrogels for sulfate ions was higher in the acidic environment. The adsorption kinetics and isothermal parameters of the hydrogels were further investigated. The adsorption kinetics of hydrogels on sulfate ions followed more of a pseudo-secondary model, indicating that the rate control of the adsorption process was a chemical reaction process. According to the fitted calculations, the adsorption reaction process is more applicable to the Langmuir isotherm model to describe the adsorption process, which indicates that the adsorption of sulfate ions is monolayer. Adsorption–desorption experiments had shown that the hydrogels had excellent reusability. In summary, the results of this study indicate that the polyhydrogel system (HEMA–NVP–MCAEM) is advantageous in the removal of anionic sulfate ions from wastewater.

## Figures and Tables

**Table 1:** The pH_PZC_ value of HNM-1, HNM-2, HNM-3, HNM-4, and HNM-5.

	HNM-1	HNM-2	HNM-3	HNM-4	HNM-5
pH_PZC_	7.59	6.73	6.68	6.66	6.61

**Table 2:** Adsorption kinetic model parameters and correlation coefficients (R^2^) for SO_4_^2−^ adsorption by HNM-4.

q_e,exp_ (mg/g)	Pseudo−First−Order Equation			Pseudo−Second−Order Equation		
	q_e,Cal_ (mg/g)	k_1_ (min^−1^)	R^2^	q_e,Cal_ (mg/g)	k_2_ (g·mg^−1^·min^−1^)	R^2^
21.53	5.138	−0.00935	0.7318	21.79	0.0459	0.9989

**Table 3:** Adsorption isotherm model parameters and correlation coefficients (R^2^) for SO_4_^2−^ adsorption by HNM-4.

Equation	Parameter 1	Parameter 2	R^2^
Langmuir	K_l_ = 0.0690	Q_max_ = 23.960	0.993
Freundlich	K_f_ = 3.913	N = 0.346	0.849

**Table 4:** The comonomer feed composition for synthesizing copolymer hydrogels.

Sample	HEMA	NVP	MCAEM
	(g)	(g)	(g)
HNM-1	4.75	4.75	0.00
HNM-2	4.75	4.75	2.00
HNM-3	4.75	4.75	3.00
HNM-4	4.75	4.75	4.00
HNM-5	4.75	4.75	5.00

## Data Availability

Not applicable.

## Sample Availability

Samples of the compounds are not available from the authors.
